# Theta event-related synchronization is a biomarker for a morbid effect of alcoholism on the brain that may partially resolve with extended abstinence

**DOI:** 10.1002/brb3.95

**Published:** 2012-10-05

**Authors:** Casey S Gilmore, George Fein

**Affiliations:** 1 Neurobehavioral Research, Inc.Honolulu, Hawaii; 2 Department of Psychology, University of HawaiiHonolulu, Hawaii

**Keywords:** Abstinent, alcoholism, biomarker, EEG, event-related synchronization, theta, time-frequency

## Abstract

Analyzing the induced (non-stimulus-phase-locked) EEG activity elicited by targets in a three-condition visual oddball task, Fein and colleagues have shown increased theta band event-related synchronization (ERS) in two different samples of long-term abstinent alcoholics (LTAA) compared with age- and gender-comparable controls. The theta ERS effect in alcoholics was also shown to be independent of, and opposite in direction to, the reduced amplitude evoked (stimulus-phase-locked) activity typically found in alcoholics and those at genetic risk of developing alcoholism. This study extends these findings by applying time-frequency analysis to target stimulus event-related EEG to compare evoked and induced theta activity in 43 LTAA and 72 nonalcoholic controls with a group of 31 alcoholics who just recently initiated abstinence from alcohol (between 6- and 15-week abstinent; referred to as short-term abstinent alcoholics, STAA). Results demonstrated that (1) evoked theta power was reduced to the same degree in STAA and LTAA compared with nonalcoholic control participants, while (2) induced theta activity, measured by theta ERS, was increased in both STAA and LTAA relative to controls, but was also increased in STAA relative to LTAA. The STAA and LTAA groups did not differ on measures of alcohol use severity or family history of alcohol problems. These results, coupled with previous findings that show a relationship between stronger theta ERS and increased memory load and attention allocation, suggest that increased theta ERS may be a biomarker for a detrimental effect of chronic alcohol abuse on the brain – a detriment that may recover, at least partially, with extended abstinence.

## Introduction

Fein and colleagues (Andrew and Fein 2010b; Gilmore and Fein 2012) have recently reported increased event-related synchronization (ERS) of induced theta activity in two different samples of long-term abstinent alcoholics (LTAA), compared to age- and gender-comparable controls in a three-condition visual oddball task. Event-related oscillatory activity, extracted with time-frequency (TF) methods, represents frequency-specific event-related changes in the ongoing EEG, and is distinguished by whether they are phase locked to the stimulus (Kalcher and Pfurtscheller 1995; Pfurtscheller and Lopes da Silva 1999). TF activity that is phase locked to the stimulus is referred to as evoked oscillations, while non-stimulus-phase-locked activity is termed induced oscillations (Klimesch et al. 1998). Theta ERS is an increase in induced theta (3–7 Hz) power relative to prestimulus values (Kalcher and Pfurtscheller 1995; Pfurtscheller and Lopes da Silva 1999).

These theta ERS findings in LTAA represent a novel and intriguing extension of the analysis of electrophysiological data recorded during simple visual target detection tasks that have previously shown differences in the average response to target stimuli between control participants and alcoholics (as well as those with other externalizing spectrum disorders; see, e.g., Porjesz et al. 2005 for a review). In examining induced theta ERS, Fein and colleagues (Andrew and Fein 2010b; Gilmore and Fein 2012) analyzed the part of the data that was considered noise in previous work: the non-stimulus-phase-locked EEG data that is typically averaged out while extracting the phase-locked, or evoked, event-related potentials and oscillations (ERPs and EROs, respectively). Furthermore, Fein and colleagues (Andrew and Fein 2010b; Gilmore and Fein 2012) found that greater theta ERS in alcoholics is independent of and opposite in direction to the classic finding of lower amplitude P3b (and lower evoked theta power) in alcoholics (e.g., Begleiter et al. 1984; Iacono et al. 2003; Fein and Chang 2006; Jones et al. 2006; Rangaswamy et al. 2007). In other words, the theta ERS effect is dissociable from the endophenotypic effects associated with the evoked measures.

Studies using healthy, nonclinical participants have shown that stronger theta ERS is associated with increased memory load and allocation of attention to task demands (Klimesch 1996; Burgess and Gruzelier 1997; Doppelmayr et al. 2000; McEvoy et al. 2001; Missonnier et al. 2006; Deiber et al. 2007). While these cognitive processes have been shown to be affected by alcohol use/abuse (Nixon and Glenn 1995; Ratti et al. 1999; Beatty et al. 2000; Schmidt et al. 2005), aspects of these processes have also been shown to at least partially resolve after long-term abstinence from alcohol (Fein and McGillivray 2007; Fein et al. 2010). Greater theta ERS in LTAA suggests that LTAA may need to engage working memory and attentional processes more strongly than do control participants in order to successfully perform the target detection task. The difference in brain activity, indexed by larger theta ERS in LTAA compared with controls, suggests that increased theta ERS may be a biomarker for a morbid effect of alcohol abuse on brain function.

While we have shown the theta ERS effect in two independent groups of LTAA, this does not establish that the effect reflects a morbid effect of alcoholism on brain function that at least partially persists into long-term abstinence. The present study compared evoked and induced theta activity in a group of alcoholics who just recently initiated abstinence from alcohol (between 6- and 15-week abstinent; referred to as short-term abstinent alcoholics, STAA) to the LTAA (>18-month abstinent) and nonalcoholic control (NAC) groups from Gilmore and Fein (2012). If the magnitude of the theta ERS decreases as a function of length of abstinence with no other concomitant differences between groups (e.g., in severity of alcohol use or in family history density of alcohol problems), it would lend more evidence to the assertion that increased theta ERS is a biomarker for an effect of alcohol use/abuse on the brain, and that this effect at least partially resolves with extended abstinence. We hypothesized that (1) evoked theta power would be reduced to the same degree in both STAA and LTAA compared with NAC, given evoked theta's role as a putative marker for the genetic vulnerability to alcoholism (which should not differ as a function of length of abstinence), but that (2) like LTAA, the STAA group would show increased induced theta ERS, but to a greater degree, that is, the magnitude of the theta ERS would be greater in STAA (given their recent initiation of abstinence from alcohol) compared with both LTAA (who have orders of magnitude longer abstinence than STAA) and controls.

## Methods

### Participants

The study sample comprised 43 LTAA (19 females, mean age = 49.0 years, SE = 0.8), who had abstinence durations ranging between 1.5 and 32.2 years (median = 3.5 years, mean = 7.6 years, SE = 1.2), 31 STAA (11 females, mean age = 49.1 years, SE = 1.3), with abstinence durations ranging between 6.0 and 14.4 weeks (median = 9.9 weeks, mean = 9.9 weeks, SE = 0.5), and 72 NAC (35 females, mean age = 48.7 years, SE = 0.8). Groups did not differ on age, *F*(2, 143) = 0.05, *P* = 0.95, nor on proportion of male and female participants in each group, χ^2^(2, *N* = 146) = 1.52, *P* = 0.47. Participants were recruited through postings at university campuses, bulletin boards, Craigslist, community and health centers, alcoholic anonymous (AA) meetings, and subject referrals. All alcoholic participants had attended AA sessions and/or other alcohol treatment programs.

Inclusion criteria for LTAA were as follows: (1) met lifetime *DSM-IV-R* (American Psychiatric Association 2000) criteria for alcohol dependence, (2) did not meet criteria for dependence or abuse of any other drug (other than nicotine or caffeine), and (3) were abstinent from alcohol and other drugs of abuse for at least 18 months. For STAA, the inclusion criteria were as follows: (1) met lifetime *DSM-IV-R* and current (within the past 12 months) criteria for alcohol dependence, (2) did not meet criteria for dependence or abuse of any other drug (other than nicotine or caffeine), and (3) were abstinent from alcohol and other drugs of abuse for a minimum of 6 weeks and a maximum of 15 weeks. NAC participants responded to advertisements for light/nondrinkers and met inclusion criteria if they had a lifetime drinking average of less than 30 standard drinks per month, with no periods of drinking more than 60 drinks per month, and no lifetime history of alcohol and substance abuse or dependence (other than nicotine and caffeine).

Exclusion criteria for all groups were as follows: (1) lifetime or current diagnosis of schizophrenia or schizophreniform disorder using the computerized Diagnostic Interview Schedule (c-DIS) (Bucholz et al. 1991; Levitan et al. 1991; Erdman et al. 1992), (2) significant history of head trauma or cranial surgery, (3) history of significant neurological disease, (4) history of diabetes, stroke, or hypertension that required emergent medical intervention, (5) laboratory evidence of hepatic disease, or (6) clinical evidence of Wernicke–Korsakoff syndrome.

### Procedures

Participant screening was initially conducted by a phone interview assessing alcohol use/dependence, use/dependence of other drugs, medical history, and mental health history. All participants were fully informed of the study's procedures and aims, and signed consent forms prior to participation. NAC subjects were asked to abstain from consuming alcohol for at least 24 h prior to any laboratory visit. A breathalyzer test (Intoximeters, Inc., St. Louis, MO) was administered, and a blood alcohol concentration of 0.000 was required of all participants in all sessions. A rapid screen test (Oral Fluid Drug Screen, Innovacon, San Diego, CA) was administered to all participants, and a negative result was required. Participants were compensated for their time and travel expenses upon completion of each session – $40 for each session and reimbursement for public transportation costs or mileage. Participants who completed the entire study were also given a $40 completion bonus. The compensation amounts and schedule were the same for all of the participants included in the current report.

Participants completed four sessions that each lasted between an hour and a half and 4 h, and included clinical, psychiatric, neuropsychological, electrophysiological, and neuroimaging assessments. Trained research associates administered a battery of assessments to all participants, among which is included an interview on their lifetime drug and alcohol use using the Alcohol Timeline Followback method (Sobell et al. 1979; Skinner and Allen 1982; Skinner and Sheu 1982; Sobell and Sobell 1990), a self-report drinking assessment tool in which drinkers use a timeline to report estimates of their alcohol consumption in phases of similar drinking behavior patterns. The drinker reports their estimates of the frequency (days/month), quantity (standard drinks/day), and the age range of that particular pattern of drinking behavior which makes up a drinking phase. A change in drinking behavior (e.g., an increase in quantity and/or frequency) would constitute a different drinking phase in the person's life. From the reported information, we are also able to determine the drinker's “peak” period which is defined as the phase of highest alcohol consumption exhibited by the drinker. This assessment yielded these alcohol consumption measures: Alcohol Peak Dosage (standard drinks per month during the course of the peak drinking phase), Alcohol Peak Use (Peak dosage × Length in days of the peak drinking phase), Alcohol Lifetime Dosage (standard drinks per month during active drinking periods over the person's life, excluding periods of sobriety), and Alcohol Lifetime Use (Lifetime dosage × Length in days of active drinking periods over the person's life, excluding periods of sobriety). Also administered is the Family Drinking Questionnaire (Mann et al. 1985), which assesses Family History Density, a measure of the proportion of first-degree relatives who had alcohol problems. The three-condition oddball task (see below) was conducted during the EEG session on the third day.

### Experimental paradigm

All stimuli were presented on a computer monitor using the E-prime software system (Psychology Software Tools Inc., Pittsburgh, PA). Stimuli were presented on a black screen for 200 msec, followed by a delay varying between 1000 and 1100 msec before the next stimulus. Three different types of visual stimuli were presented: (1) standard nontarget stimuli, a small hollow white square; (2) target stimuli, a small white X; and (3) novel rare nontarget stimuli, different shapes of various colors. Participants responded with the index finger of their dominant hand and were instructed to press a response box button only when they saw target stimuli. Stimuli were presented in a predetermined order, with standard stimuli appearing 210 times, target stimuli appearing 35 times, and rare nontarget stimuli appearing 35 times over approximately 6.5 min. Each participant was shown an example of the target stimulus before the task began.

### EEG acquisition and analysis

EEG was acquired using a 64-channel system that used the SynAmps2 amplifier and Scan 4.3 acquisition software (Compumedics Neuroscan Inc., Charlotte, NC). The EEG signal was referenced to an electrode located between Cz and CPz for online recording, and then re-referenced to the right ear offline. The ground electrode was placed 8 cm above the nasion. Electrode impedances were maintained below 10 kOhms. The SynAmps2 amplifier had a fixed range of ±333 μV sampled with a 24-bit A/D converter where the least significant bit was 0.019 μV. The sampling rate was 250 samples/sec.

EEG recordings were processed offline using the Edit program in Scan 4.3 (Compumedics Neuroscan, Inc., Charlotte, NC). Artifacts from eye movements were removed using the ocular artifact reduction algorithm (ARTCOR procedure) in Scan 4.3. Data were then band-pass filtered between 0.5 and 30 Hz using a zero-phase lag filter at 48 dB/octave. Stimulus-locked epochs for the target condition were extracted for all instances where there was a correct behavioral response. Trials consisted of 1800 msec of data, including a 500-msec prestimulus baseline. Any epochs with voltages beyond the range of ±75 μV were rejected as artifacts and excluded from further processing.

In addition to the visual oddball task, 5 min of baseline eyes open resting EEG was recorded at the start of the session.

### Time-frequency measures

TF measures were computed using the Cohen's class binomial reduced interference distribution (RID) transform (see Williams 1996; Bernat et al. 2005 for more detailed descriptions), the advantage of which is that it provides a uniform resolution across the TF surface. TF representations were created using the entire 1800-msec epoch to minimize edge effects.

TF measures were extracted in two ways: (i) for evoked theta activity, decompositions were performed using averaged ERP data, measuring brain activity phase locked to the stimuli, while attenuating nonphase-locked (i.e., induced) activity, and (ii) for induced theta activity, the trial-averaged ERP waveform was first subtracted from each single trial data, with the residual being transformed to the TF domain. The resultant single trial TF surfaces were then averaged across trials to produce a TF representation of the event-related nonphase-locked TF activity. With these methods, one evoked TF representation and one induced TF representation were produced for each electrode site for each subject. To confirm the non-stimulus-phase-locked nature of the induced theta activity, intertrial coherence (ITC), a measure of the extent to which phase locking occurs across trials, was also calculated for induced theta at each electrode for each subject.

Based on visual inspection of the grand-averaged evoked and induced TF representations, poststimulus TF regions of interest (TFROIs), encompassing the theta-frequency band, were selected. The TF power was averaged within each of these TFROIs. For induced activity, in addition to the poststimulus TFROI, a corresponding prestimulus TFROI was also selected, covering the same frequency range as the poststimulus TFROI, but with a time window occurring prior to target stimulus onset. This prestimulus TFROI was utilized as a reference for comparing event-related changes in poststimulus power, that is, ERS/event-related desynchronization, which was computed as the log ratio of the poststimulus power to the prestimulus power (see Andrew and Fein 2010b, for a more detailed description).

To examine resting theta power, the resting EEG data were first corrected for ocular artifacts, then divided into 1024-msec half-overlapping epochs (i.e., the first 512 msec of each epoch overlapped with the last 512 msec of the preceding epoch). Epochs with EOG amplitude >75 μV were eliminated from further processing. Fourier transform-based spectral estimation, using Welch's periodogram method, was then applied to each artifact-free epoch using a Hamming window, resulting in power spectra with 1-Hz resolution. The mean absolute power within the same theta-frequency range used in the evoked and induced TF analyses (3–6 Hz, see below) was then calculated for each electrode site. Because the distribution of theta power was skewed, the data were log transformed.

### Statistical analysis

All statistical analyses were performed using SPSS (SPSS Inc., Chicago, IL). The measures submitted to statistical analysis were (1) evoked theta power (log-transformed) averaged over electrodes Pz and CPz and (2) induced theta activity (theta ERS) averaged over electrodes FCz and Cz. These electrodes were those within which each of the measures was found to be maximal, both in the current study and in previous reports (e.g., Jones et al. 2006; Andrew and Fein 2010a,b; Gilmore and Fein 2012), and thus were considered those best characterizing each of the measures. The evoked and induced theta measures, and ITC (also averaged over FCz and Fz), were submitted, separately, to univariate analyses of variance (ANOVAs) with between-subjects factor group (NAC, LTAA, and STAA). Given our a priori hypotheses (that for evoked theta, power would be reduced to the same degree in both STAA and LTAA compared with NAC; for induced theta, the magnitude of the theta ERS would be greater in LTAA vs. NAC, and greater in STAA compared with both LTAA and NAC), pairwise comparisons between each group within the evoked and induced theta univariate ANOVAs were planned. Tukey's HSD test was used to test the significance of these multiple comparisons while maintaining the α = 0.05 experiment-wise error rate. To investigate any group differences in induced theta ERS that might be related to the value of the prestimulus theta power, an analysis of covariance (ANCOVA), with the mean (log-transformed) power within the prestimulus TFROI as the covariate, between-subjects factor group, and dependent variable induced theta, was performed, along with follow-up pairwise comparisons between NAC, STAA, and LTAA.

Independent samples *t*-tests were used to evaluate (at *P* < 0.05) any differences between LTAA and STAA with regard to their severity of, and genetic predisposition to, alcohol use/abuse. The two groups were compared on the measures: Alcohol Peak Dosage, Alcohol Peak Use, Alcohol Lifetime Dosage, Alcohol Lifetime Use, Lifetime Alcohol Dependence and Alcohol Abuse symptom counts, and Family History Density.

## Results

### Behavioral results

A univariate ANOVA showed that there was no significant group difference on accuracy of responding to target stimuli (*F*(2, 140) = 2.80, *P* = 0.07). Group means (±SE) for accuracy were (of 35 total targets) NAC: 34.55 ± 0.14, LTAA: 34.02 ± 0.18, and STAA: 34.39 ± 0.21. An ANOVA revealed a significant group main effect for reaction time for pressing the response box button to targets (*F*(2, 140) = 3.52, *P* = 0.03). Tukey's HSD post hoc tests showed that NAC (mean = 422.20 msec ± SE = 7.78) responded slightly faster on average than did LTAA (455.22 msec ± 9.85), while STAA (439.59 msec ± 11.60) did not differ from either NAC or LTAA.

### Time-frequency measures

**Figure 1:**
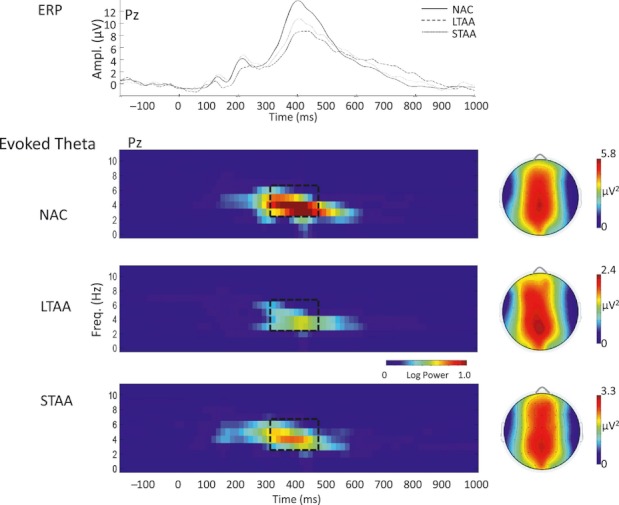
At top are the grand-averaged ERPs at electrode Pz for the target stimulus for NAC, LTAA, and STAA. Below are the grand-averaged evoked (stimulus-phase-locked) time-frequency representations (TFR), band-pass filtered in the theta band (3–8 Hz) to accentuate the relevant activity, at electrode Pz for the target stimulus for NAC, LTAA, and STAA, together with the topographic maps for the mean activity within the theta time-frequency region of interest (indicated by the dashed box on each TFR). The topographic maps are scaled differently so as to clearly indicate the spatial distribution of each.

The averaged evoked TF representation for each group at electrode Pz for the target stimulus is shown in Figure 1. For illustration purposes, in order to accentuate the evoked theta activity analyzed in the present study, the TF representations were filtered in the theta band (3–8 Hz). Based on visual inspection of the grand-averaged evoked TF surfaces, a theta band TFROI was selected that spanned a time range of 325–450 msec and a frequency range of 3–6 Hz (indicated by a box overlaid on the evoked TF surfaces). Figure 1 also shows topographic maps for the mean activity within the TFROI for each group. The grand-averaged ERPs for each group, that is, the evoked data submitted to TF analyses, are also shown at the top of Figure 1.

**Figure 2:**
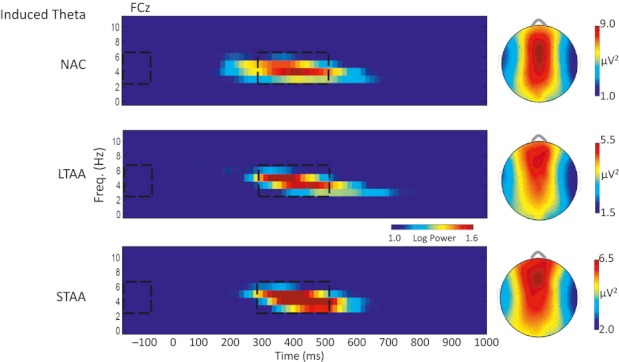
Grand-averaged induced (non-stimulus-phase-locked) time-frequency representations (TFR), band-pass filtered in the theta band (3–8 Hz) to accentuate the relevant activity, at electrode FCz for the target stimulus for NAC, LTAA, and STAA. The poststimulus and prestimulus theta TFROIs are indicated by dashed boxes on the TFRs. Topographic maps for the mean activity within the poststimulus theta TFROI for NAC, LTAA, and STAA are shown.

The averaged induced TF representation for each group at electrode FCz for the target stimulus is shown in Figure 2. These TF representations, also filtered in the theta band to accentuate the relevant activity, show an ERS of theta activity occurring between about 200 and 600 msec. Based on visual inspection of the grand-averaged induced TF surfaces, a theta poststimulus TFROI was selected that spanned a time range of 250–475 msec and a frequency range of 3–6 Hz (indicated by a box overlaid on the induced TF surfaces). The corresponding prestimulus reference TFROI (also identified by a box on the TF surface) had the same frequency range, with a time range of −200 to −95 msec. Figure 2 also shows topographic maps for the mean activity within the poststimulus TFROI for each group. ITC values for statistical analyses were obtained by averaging ITC within the same poststimulus TFROI as the induced theta.

Group means (±SE) for evoked and induced theta were as follows: evoked theta (log-transformed power; NAC: 0.61 ± 0.06, LTAA: 0.26 ± 0.07, STAA: 0.25 ± 0.09) and induced theta (theta ERS, calculated as the log ratio of poststimulus/prestimulus power; NAC: 0.15 ± 0.03, LTAA: 0.28 ± 0.04, STAA: 0.44 ± 0.05). In order to understand the shared and/or unique variance between the evoked and induced theta measures, first at an overall level (without regard to alcohol vs. control group differentiation), we performed correlations (Pearson's *r*) between them, collapsing across group. Evoked and induced theta were not associated, *r* = −0.01, *P* = 0.96, showing that they are independent measures, sharing no significant variance.

### Evoked theta

For evoked theta, the main effect of group was significant (*F*(2, 143) = 10.17, *P* < 0.001, η_p_^2^ = 0.12). The Tukey's HSD test indicated that while mean evoked theta power for both the STAA and LTAA groups was significantly smaller than that of NAC (both *P* < 0.002, Cohen's *d* effect sizes = 0.76 and 0.74, respectively), the STAA and LTAA groups did not significantly differ from each other (*P* = 0.99, *d* = 0.02).

### Induced theta

Induced theta ERS also showed a significant main effect of group (*F*(2, 143) = 14.01, *P* < 0.001, η_p_^2^ = 0.16). Tukey's HSD revealed that all pairwise differences among group means were significant (all *P* < 0.03). These results show (1) that theta ERS was larger in both STAA and LTAA compared with controls (Cohen's *d* = 1.13 and 0.50, respectively) and (2) the magnitude of this enhancement (vs. controls) was greater in STAA than in LTAA (i.e., the theta ERS effect was smaller as a function of length of abstinence; *d* = 0.64). ITC values were very low (mean ITC ± SE: NAC: 0.078 ± 0.003, STAA: 0.079 ± 0.004, LTAA: 0.088 ± 0.004), indicating that the induced theta activity was not consistently phase locked to the stimulus, and did not differ between groups (*F*(2, 143) = 2.63, *P* = 0.08).

To investigate any relationship between these induced theta group effects and prestimulus levels of theta power during the task, first, a univariate ANOVA was performed with group as the between-subjects factor and the dependent measure mean prestimulus theta power (log-transformed power averaged within the prestimulus TFROI). There was a main effect of group (*F*(2, 143) = 14.96, *P* < 0.001, η_p_^2^ = 0.17). Post hoc Tukey's HSD tests showed that while NAC (mean = 0.69 ± SE = 0.03) and LTAA (0.66 ± 0.03) did not differ from each other (*P* = 0.78, *d* = 0.13), STAA (0.45 ± 0.04) had lower task prestimulus theta power than both NAC and LTAA (both *P* < 0.001, *d* = 1.15 and 1.02, respectively). Next, an ANCOVA on induced theta with task prestimulus power as the covariate showed a main effect of group (*F*(2, 142) = 4.94, *P* = 0.008, η_p_^2^ = 0.07). Follow-up pairwise comparisons on the groups' adjusted means revealed, however, that while induced theta ERS in both STAA and LTAA were still larger than NAC (both *P* ≤ 0.01; *d* = 0.61 and 0.49, respectively), STAA and LTAA no longer differed from each other (*P* = 0.66, *d* = 0.11). Finally, to examine whether this group difference in task prestimulus theta power may be related to general baseline levels of theta activity, resting EEG theta power was compared between groups via a univariate ANOVA. Resting EEG theta power (log-transformed), group means (±SE) were NAC: 0.71 ± 0.03, LTAA: 0.71 ± 0.04, STAA: 0.76 ± 0.05. Levels of resting theta power did not significantly differ between groups (*F*(2, 140) = 0.46, *P* = 0.63, η_p_^2^ = 0.007).

### Differences in severity of alcohol use/abuse between STAA and LTAA

*T*-tests performed using measures of alcohol use/abuse severity and family history of alcohol problems revealed that the STAA and LTAA groups did not differ on any measure. Group means and *t*-values are shown in Table 1.

**Table 1:** Group means (SE) and results of *t*-tests comparing STAA and LTAA on measures of alcohol use/abuse severity and family history of alcohol problems

	Mean (SE)		
		
	STAA	LTAA	*t* (df = 72)^1^
Alcohol Peak Dosage^1^	367.65 (80.43)	426.62 (45.26)	0.68
Alcohol Peak Use^1^	40,651.55 (9430.50)	28,851.95 (4768.88)	1.21
Alcohol Lifetime Dosage^1^	200.16 (42.92)	232.91 (28.43)	0.66
Alcohol Lifetime Use^1^	69,256.52 (13,240.30)	69,945.38 (10,514.48)	0.04
Lifetime Alcohol Dependence symptom count	6.10 (0.20)	5.79 (0.16)	1.19
Lifetime Alcohol Abuse symptom count	2.84 (0.23)	2.86 (0.18)	0.08
Family History Density	0.34 (0.06)	0.30 (0.04)	0.48

1 No comparisons were significantly different (all *P* > 0.05). Alcohol Peak Dosage, standard drinks per month during the course of the peak drinking phase; Alcohol Peak Use, Peak dosage × Length in days of the peak drinking phase; Alcohol Lifetime Dosage, standard drinks per month during active drinking periods over the person's life, excluding periods of sobriety; Alcohol Lifetime Use, Lifetime dosage × Length in days of active drinking periods over the person's life, excluding periods of sobriety.

## Discussion

This study evaluated differences between STAA, LTAA, and age- and gender-comparable controls in evoked and induced theta TF activity in response to targets in a simple, visual oddball task. Results demonstrated that (1) evoked theta power was reduced to the same degree in STAA and LTAA compared with NAC participants, while (2) induced theta activity, measured by theta ERS, was increased in both STAA and LTAA relative to controls, but was also increased in STAA relative to LTAA.

Controlling for the between group variance in task prestimulus theta power (which was lower in STAA compared with NAC and LTAA), theta ERS, while still higher in alcoholic groups versus controls, did not differ between STAA and LTAA. Moreover, task prestimulus theta power was not a proxy for baseline theta activity. Thus, the STAA–LTAA group differences in theta ERS is largely attributable to lower task prestimulus theta activity in STAA relative to LTAA, possibly reflecting group differences in task-related attention and working memory-related processes (see below for discussion).

Our findings in three different samples of adult alcoholics (in two independent samples of LTAA in Andrew and Fein 2010b; Gilmore and Fein 2012; and a sample of STAA in this study) are consistent with the proposition that larger theta ERS is an effect of alcohol exposure on the brain. Theta ERS decreased with duration of abstinence (although it was still higher relative to controls even with multiyear abstinent-treated alcoholics). The LTAA and STAA in this study did not differ with regard to their alcohol burden; the groups were comparable in their lifetime and peak severity of use and in their lifetime symptom counts of alcohol dependence and abuse. Furthermore, LTAA and STAA did not differ in their family history density of alcohol problems – a finding that is also consistent with our results showing that the two groups did not differ in the magnitude of the reduction of evoked theta power nor in levels of resting EEG theta power relative to controls, both of which measures have been shown to be genetically influenced (e.g., Tang et al. 2007a,b; Zlojutro et al. 2011). In total, these findings suggest that higher than normal theta ERS in a simple target detection task is an effect of chronic alcohol abuse on the brain that may recover (albeit incompletely) with extended abstinence.

It is unclear exactly what larger theta ERS is signaling in alcoholics. Prior research shows that theta ERS is associated with working memory and attentional processes (Klimesch 1996; Burgess and Gruzelier 1997; Doppelmayr et al. 2000; Krause et al. 2000; McEvoy et al. 2001; Missonnier et al. 2006; Deiber et al. 2007). The relationship between induced theta ERS and working memory and attention has been mostly studied within the context of n-back tasks. For example, both Krause et al. (2000) and Deiber et al. (2007) showed that sustained induced theta activity tends to increase with increased memory load and/or allocation of attention to task demands. These studies proposed that induced theta activity modulated by task demands likely reflects activity in cortico-hippocampal neural loops involved in task-relevant stimulus identification and retention in working memory in anticipation of further task-related requirements (Klimesch 1996; Newman and Grace 1999; Krause et al. 2000; Deiber et al. 2007).

Alcohol use/abuse has been associated with working memory and attention deficits (Nixon and Glenn 1995; Ratti et al. 1999; Beatty et al. 2000; Schmidt et al. 2005), as well as with structural and functional neural adaptations in the brain as a result of chronic alcohol exposure (Nixon 2006). For example, animal and human studies have reported associations between chronic alcoholism and neurodegeneration in the hippocampus and superior frontal cortices (Walker et al. 1980; Agartz et al. 1999; Sullivan and Pfefferbaum 2005; Nixon 2006), areas involved in the working memory and attention deficits associated with alcoholism. These cognitive and brain deficits have been shown to recover, however, following periods of abstinence from alcohol (Nixon 2006; Fein and McGillivray 2007; Fein et al. 2010). Greater theta ERS in STAA and LTAA suggests that they are engaging working memory and attentional processes to a greater extent than are control participants in order to perform the target detection task successfully, a process that may be pronounced in STAA given that their larger theta ERS is concomitant with lower (relative to NAC and LTAA) prestimulus levels of theta activity. Recent studies suggest that prestimulus levels of theta activity reflect a state of “cognitive readiness,” for example, a specific allocation of attention, to perform an upcoming task (Min and Park 2010; Min et al. 2011). Larger theta ERS, then, may reflect a compensatory mechanism for attention and/or working memory dysfunction (reflected in lower prestimulus theta) in STAA. Furthermore, given that LTAA had (1) similar levels of prestimulus theta to controls and (2) a smaller magnitude theta ERS increase, compared with controls, than did STAA, suggests that these cognitive deficits may at least partially recover after extended abstinence. Relatedly, greater theta ERS may reflect the structural and functional neural plasticity associated with the development of, and recovery from, chronic alcohol dependence.

A caveat in this study is that the task may have been too easy to show group differences in task performance (accuracy was very high and did not differ between groups). Even with successful task performance, however, the brain activity differences persisted. Thus, we hypothesize that greater theta ERS may index compensatory mechanisms in alcoholics to overcome working memory and attention deficits – deficits that may partially recover with long-term abstinence. Furthermore, given the relationship between theta ERS and task demands, this compensatory mechanism may break down as task demands increase. Given these possibilities, and given theta ERS's relationship with memory and attentional processes, future research should more systematically examine how task demands affect differences between alcoholic and control groups in theta ERS, and in prestimulus theta activity, in order to refine our understanding of alcohol- and abstinence-related brain changes.

In summary, results presented in this study support the proposition that increased theta band ERS is a biomarker for a morbid effect of alcohol use/abuse on the brain. A limitation of this study, however, is that only treated samples of abstinent alcoholics were examined. The number of alcoholics who have been in treatment or AA is only a small proportion – less than a quarter – of alcoholics in the general population (Fein and Landman 2005). Our current findings, then, cannot be generalized to the larger population of alcoholics. This limitation could be addressed by investigating the induced theta ERS effect in treatment naïve actively drinking alcoholics (TNAs), who make up the bulk of alcoholics in society. TNAs are not simply treated alcoholics observed early in the course of their illness, but rather comprise a separate population with less severe alcoholism (Fein and Landman 2005; Sclafani et al. 2008; Smith and Fein 2010; Fein and Andrew 2011). Studying TNA, then, will increase our understanding of the societal implications of theta ERS's role as a biomarker for alcohol abuse's effect on the brain.
